# The effect of silicone oil tamponade on retinal layers and choroidal thickness in patients with rhegmatogenous retinal detachment: a systematic review and meta-analysis

**DOI:** 10.1186/s40942-021-00348-y

**Published:** 2021-12-20

**Authors:** Heshmatollah Ghanbari, Farzan Kianersi, Alireza Jamshidi Madad, Alireza Dehghani, Alireza Rahimi, Awat Feizi, Afsaneh Naderi Beni

**Affiliations:** 1 Isfahan Eye Research Center, Isfahan University of Medical Sciences, Isfahan, Iran; 2 Clinical Informationist Resarch Group, Health Information Technology Research Center, Isfahan University of Medical Sciences, Isfahan, Iran; 3 Department of Biostatistics and Epidemiology, School of Health, Isfahan University of Medical Sciences, Isfahan, Iran

**Keywords:** Rhegmatogenous retinal detachment, Silicone oil, Retinal layer thickness, Choroidal thickness

## Abstract

**Background:**

To evaluate the effects of intravitreal silicone oil (SO) on the retinal and choroidal thickness in eyes with rhegmatogenous retinal detachment (RRD).

**Methods:**

A literature search was performed in Web of Science, Scopus, ProQuest, Embase, Clinical Key, Science Direct, Cochrane Library, and Springer, as well as Persian databases, including IranDoc, MagIran, SID, MOH thesis, and MOH articles until June 2020. Two reviewers independently searched and extracted the data.

**Results:**

Sixteen studies (n = 391) met the inclusion criteria. The meta-analysis showed that the SO tamponade could significantly reduce the central macular thickness (CMT) in patients with RRD as compared to gas tamponade WMD = − 14.91; 95% CI: − 22.23, − 7.60; P < 0.001, I^2^ = 71%). No significant change was found in CMT between the eye with SO tamponade (after SO removal) and the fellow healthy eye in patients with RRD (WMD = − 3.52; 95% CI: − 17.63, 10.59; I^2^ = 68.6%). Compared to the preoperative stage, the SO tamponade could significantly reduce the subfoveal choroidal thickness in patients with RRD (WMD = − 18.67, 95% CI: − 30.07, − 1.28; I^2^ = 80.1%). However, there was no significant difference in the subfoveal choroidal thickness before and after SO removal (WMD = − 1.13, 95% CI: − 5.97, 3.71; I^2^ = 87.6%).

**Conclusion:**

The SO tamponade had a significant effect on the reduction of retinal layers and the subfoveal choroidal thickness.

## Background

Retinal detachment (RD) is one of the leading causes of permanent vision loss. It can be classified as rhegmatogenous, tractional, or exudative. Rhegmatogenous retinal detachment (RRD) is the most common type of RD, occurring in one per 10,000 people each year. Retinal surgery is increasingly used for the repair of RRD, and recent developments in techniques and instruments have improved the outcomes [1, 2]. Silicone oil (SO) and gas are two of the most commonly used tamponading agents in vitreoretinal surgery, each with its own advantages and disadvantages. Although there is a consensus on what necessitates gas or SO tamponade, they can be still selected at the discretion of the surgeon or based on regional differences [3].

The long-term SO tamponade complications include secondary glaucoma, cataract, optic neuropathy, band keratopathy, and retinal toxicity [4]. Mild vision loss may occur after SO removal, usually due to postoperative cystoid macular edema (CME), hypotony, optic neuropathy, and development of epiretinal membranes; nevertheless, there are some cases of visual loss that do not have a certain cause [4–6] The possible mechanisms include macular dysfunction, mostly of ganglion and bipolar cell synapses, and optic nerve damage due to SO infiltration [5].

Optical coherence tomography shows that long-term SO tamponade may have a negative effect on the thickness of retinal layers in some patients [7, 8]. Also, some researchers have shown that SO tamponade may have a negative long-term effect on retinal microcirculation [9, 10]. Besides, in RRD patients without macular involvement, the prevalence of unexplained vision loss is higher after SO tamponade as compared to gas tamponade [8, 11].Recent studies also suggest that visual abnormalities, such as decreased foveal sensitivity, central scotoma, and macular dysfunction, may be related to SO tamponade [12, 13].

Recent studies examining the effect of retinal and choroidal layers have reported controversial results, including a study by Goker et al., which found that the thickness of the inner nuclear layer (INL) and outer plexiform layer (OPL) increased in the eyes with SO tamponade in comparison with the fellow healthy eyes [14]. However, in a study by Lee et al. on patients with RD, it was found that in the SO tamponade group, there was a marked reduction in the inner and outer retinal layer thickness compared to the gas tamponade group [15]. Moreover, a study by Karimi et al. showed that the subfoveal choroidal thickness (SFCT) was reduced in patients receiving SO tamponade [16]. Conversely, a study by Zhou et al. reported a decrease in the retinal layer thickness, while no significant change was observed in the choroidal thickness [17].

Studies have been conducted regarding the effect of SO tamponade on retinal and choroidal thickness in recent years. However, there is no systematic review of studies investigating the effect of SO tamponade on retinal layers. Therefore, in the present study, we systematically reviewed the effect of SO tamponade on the central macular thickness (CMT), different retinal layers, and choroidal thickness.

## Methods

### Search strategy

In this systematic review, we unanimously approved the main concepts using the PICO format (Table 1) Based on the PICO search format, a free search strategy was used to perform a more comprehensive and accurate search of information sources in different databases. Also, in this study, all published articles were included without any time limits. A search of the following international databases was performed until June 2020: Web of Science, PubMed, Scopus, ProQuest, Clinical Key, Science Direct, Embase, Cochrane Library, and Springer, as well as Persian databases, such as MagIran, SID, IranDoc, MOH thesis, and MOH article databases. The included studies were clinical trials, clinical interventions (before-and-after analysis), and cohorts that met the inclusion criteria after screening.

**Table 1:** Electronic database search terms

Synonyms	Keywords	
“Retinal detachment” OR “Retinal detachment surgery” OR “Rhegmatogenous retinal detachment”	Retinal detachment	Population/disease
“Silicone oil” OR “Silicone oil endotamponade” OR “Endotamponade” OR “Intravitreal silicone oil” OR “Intravitreal tamponade” OR “Intraocular tamponade” OR “Silicone oil removal”	Silicone oil tamponade	Intervention
“Gas tamponade” AND “Fellow eye”	Gas tamponade and fellow eye	Comparison
“Retinal layer thickness” OR “Retinal thickness” OR “Macular layers” OR “Macular layer thickness” OR “Central macular thickness” OR “Central retinal thickness” OR “Retinal nerve fiber layer” OR “Ganglion cell layer” OR “Inner plexiform layer” OR “Outer plexiform layer” OR “Inner nuclear layer” OR “Outer nuclear layer” OR “Rod and cone inner and outer segments (IS/OS)” OR “Inner retinal layers” OR “Outer retinal layers” OR “Choroidal thickness” OR “Subfoveal choroidal thickness”	Retinal layers	Consequences

The inclusion criterion in this systematic review was all studies investigating the effect of SO tamponade on retinal and choroidal layers in patients with RRD. There was no age, sex, magazine type, or publication limit, and all articles meeting the inclusion criteria were evaluated. The exclusion criteria were as follows: a macular hole, a macular pucker, or other maculopathies; RRD with high myopia, uveitis, glaucoma, macular edema, or postoperative endophthalmitis; traumatic RD; and other optical nerve diseases. Also, case reports and systematic reviews were excluded.

### Data extraction and quality assessment

After searching for studies based on the keywords, eligible articles were extracted from the scientific databases. The flowchart of the articles was plotted by one of our colleagues (A.R.). In the first stage, by reviewing the title and abstract of studies, a number of unrelated and duplicate studies were eliminated. In the next step, a detailed evaluation of the full-text of the remaining articles was performed. All steps were performed by two independent researchers (H.Gh. and A.R.J.), and the third researcher (F.K.) supervised and studied their findings [18–20]. The following data were also extracted from eligible studies by two researchers (H.Gh. and A.R.J.): the first author’s name, publication date, sample size, age, the duration of SO tamponade dressing, time spent after SO removal, type of RRD (on/off), CMT, retinal layer segmentation thickness, and SFCT. The quality of studies was also assessed by completing the CONSORT checklist for clinical trials and STROBE checklist for observational studies; based on the score that each study received, we decided to remove or include it.

### Data synthesis and statistical analysis

Two rounds of data analysis were conducted. For all included studies, whether they had a control group or not, the effect of SO was evaluated by comparing the outcomes before and after the intervention. Also, for studies with a control group, the mean difference in the outcomes before and after the intervention was calculated for each group and compared between the SO group and the control group. Moreover, the effect size was estimated as the weighted mean difference (WMD) or standardized mean difference (SMD) and 95% confidence interval (95% CI). Heterogeneity between studies was evaluated by Cochran’s Q and I-square (I^2^) tests; I^2^ above 50% was regarded as substantial heterogeneity [21].

To calculate the effect sizes and their corresponding 95% CIs, a fixed effects model was adopted when heterogeneity was low. On the other hand, a random effects model was used in case of moderate or high heterogeneity, considering the between-study variations in the DerSimonian and Laird method [22]. To explore the source of heterogeneity, a meta-regression analysis was performed to evaluate the confounding role of age and duration of SO tamponade. Besides, sensitivity analyses were performed to evaluate the extent to which inferences might be related to a particular study. Publication bias was also examined by the visual inspection of funnel plots [23]. Moreover, a statistical evaluation of funnel plot asymmetry was performed by Egger’s and Begg’s regression tests [23]. Both the funnel plot and the Egger and Begg tests do not have good power to assess publication bias in meta-analysis with less than 10 studies. It tried to do the bias analysis, but it doesn’t have enough studies. When there was publication bias, the trim-and-fill method was applied to detect the contribution of bias to the overall effect. Statistical analyses were performed in Stata version 11 (Stata Corp., College Station, TX, USA). P-values less than 0.05 were considered statistically significant.

## Results

### Search results and study characteristics

**Fig. 1:**
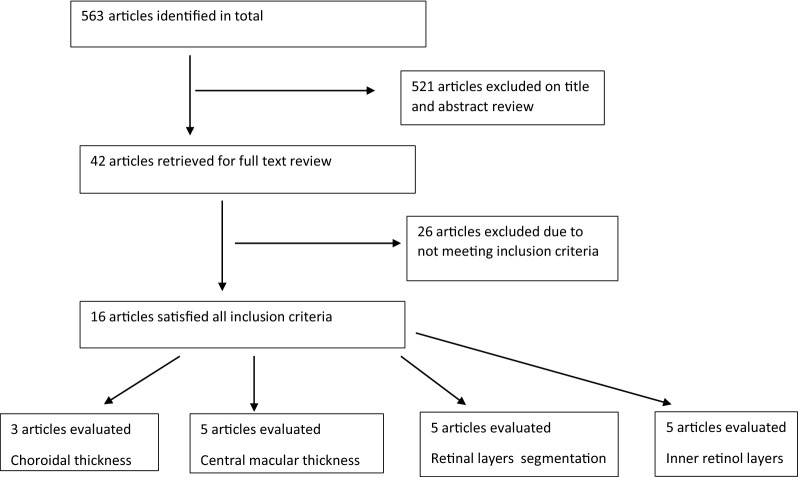
The results of the literature search strategy. The flow diagram depicts the screening process of articles and the number of exclusions

As shown in Table 2, the initial search yielded a total of 563 publications. Based on the inclusion and exclusion criteria, 16 articles were found to be eligible: five studies had arms only evaluating the CMT [24–29], three studies only evaluating the inner retinal layer thickness [7, 8, 28]. five studies evaluating both CMT and retinal layer segmentation thickness [14, 15, 17, 30, 31], and three studies only evaluating the SFCT [16, 32, 33].In the search of Persian databases, no relevant studies were found. Figure 1 presents the flow chart of the selection process used to identify studies.

**Table 2:** The general characteristics of the included studies in the systematic review

First author	Publication year	Quality of study	Age (mean)	Sample size	Type of RRD (on/off)	Duration of SO tamponade (month)	Time after SO removal (month)	CMT	Inner retinal layer thickness	Retinal layer segmentation thickness	SFCT
Hostovsky [24]	2020	Good	57.7	SO = 20	Off	1.5 -2.5	6	*			
Rabina [27]	2019	Excellent	56.1	SO = 41	On	3- 6	1	*			
Takkar [25]	2017	Good	39.6	SO = 32	Off	3–6	6–9	*			
Roohipoor [26]	2020	Excellent	54	SO = 45	Off	3	–	*			
Kheir [29]	2018	Good	48.1	SO = 10	On	3	9	*			
Tode [28]	2016	Fair	61	SO = 15	On	1.5	36		*		
Caramoy [34]	2014	Fair	67	SO = 9	On	5	–		*		
Christensen [8]	2011	Fair	51	SO = 9 Gas = 7	On	5	46		*		
Inan [31]	2016	Good	60.7	SO = 28 Gas = 30	Off	4	8	*		*	
Goker [14]	2017	Good	56.35	SO = 20 Gas = 16	Off	4	2	*		*	
Lee [15]	2017	Good	46.83	SO = 33 Gas = 31	On	3.5	6	*		*	
Purtskhvanidze [30]	2017	Good	59.3	SO = 20 Gas = 20	Off	5.5	35	*		*	
Zhou [17]	2020	Good	53.86	SO = 7 Gas = 14	On	4	–	*		*	
Mirza [32]	2018	Fair	60.4	SO = 24	Off	3–6	1				*
Karimi [16]	2018	Good	52	SO = 60	Off	6–9	3				*
Odrobino [33]	2016	Good	62.44	SO = 18	Off	6	-				*

A scoring scale for this study based on Strobe modified (≥ 85 = Excellent, 70 to < 85 = Good, 50 to < 70 = Fair, < 50 = poor)

Out of 16 articles reviewed, the findings of 11 articles were meta-analyzed [13–18, 20, 22–25]. The findings of the other five articles are presented only quantitatively due to the lack of necessary information in the statistical analysis [5, 6, 19, 21, 26]. Of the selected articles, 13 studies were retrospective, two were prospective, and one was cross-sectional. A total of 391 patients were included in these studies, and their mean age was 55.36 years. Table 2 summarizes the characteristics of the included studies in this systematic review.

## Meta-analysis and data synthesis

### Effect of SO tamponade on the retinal layer thickness

There are two types of articles on the effect of SO tamponade on the retinal layers. Some studies only examined the effect of SO tamponade on the CMT or inner retinal layers, while some studies, in addition to CMT, examined the effect of SO tamponade separately on the retinal layers.

### Effect of SO tamponade on CMT: (Fig. 2a–o)

**Fig. 2:**
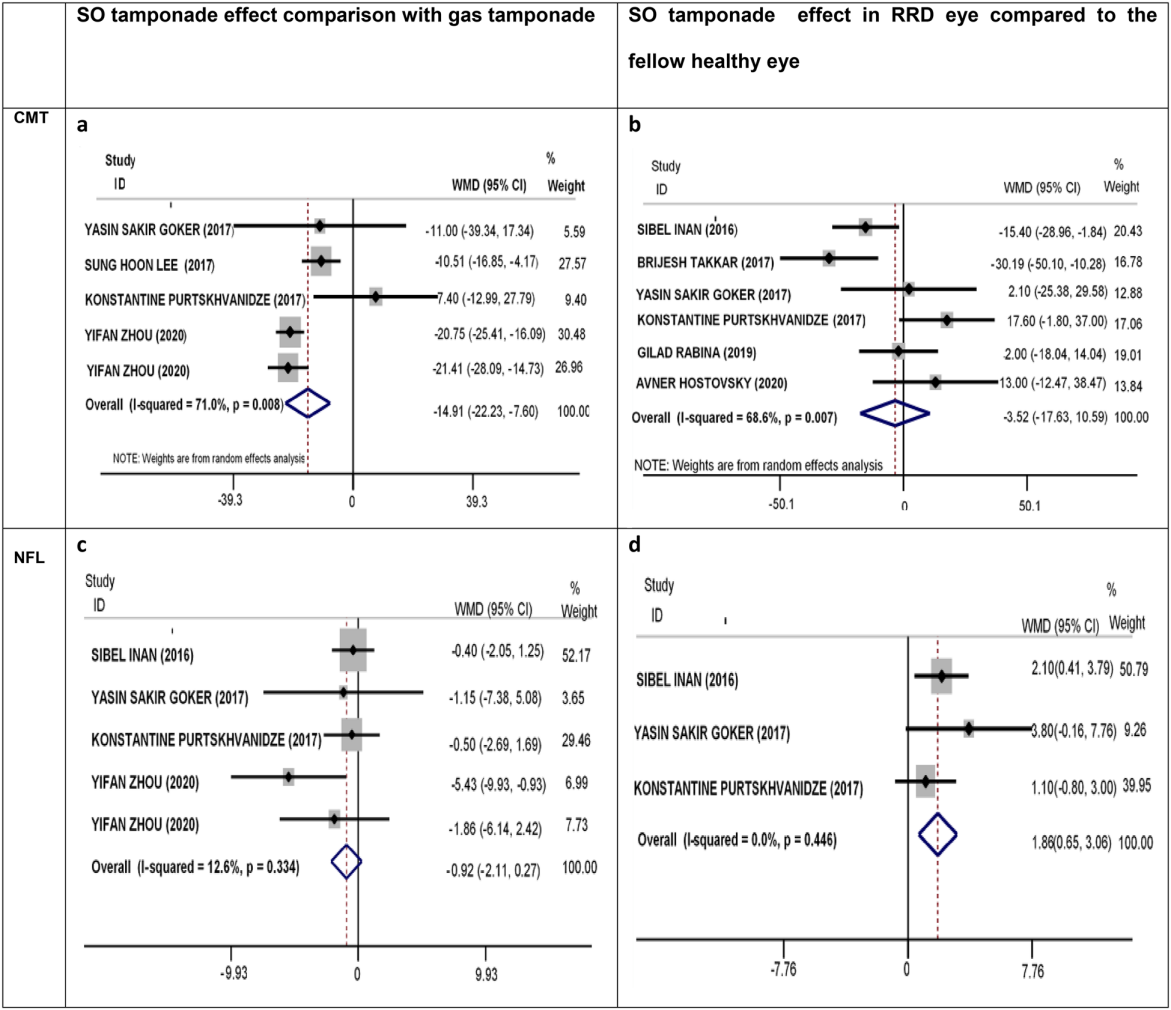

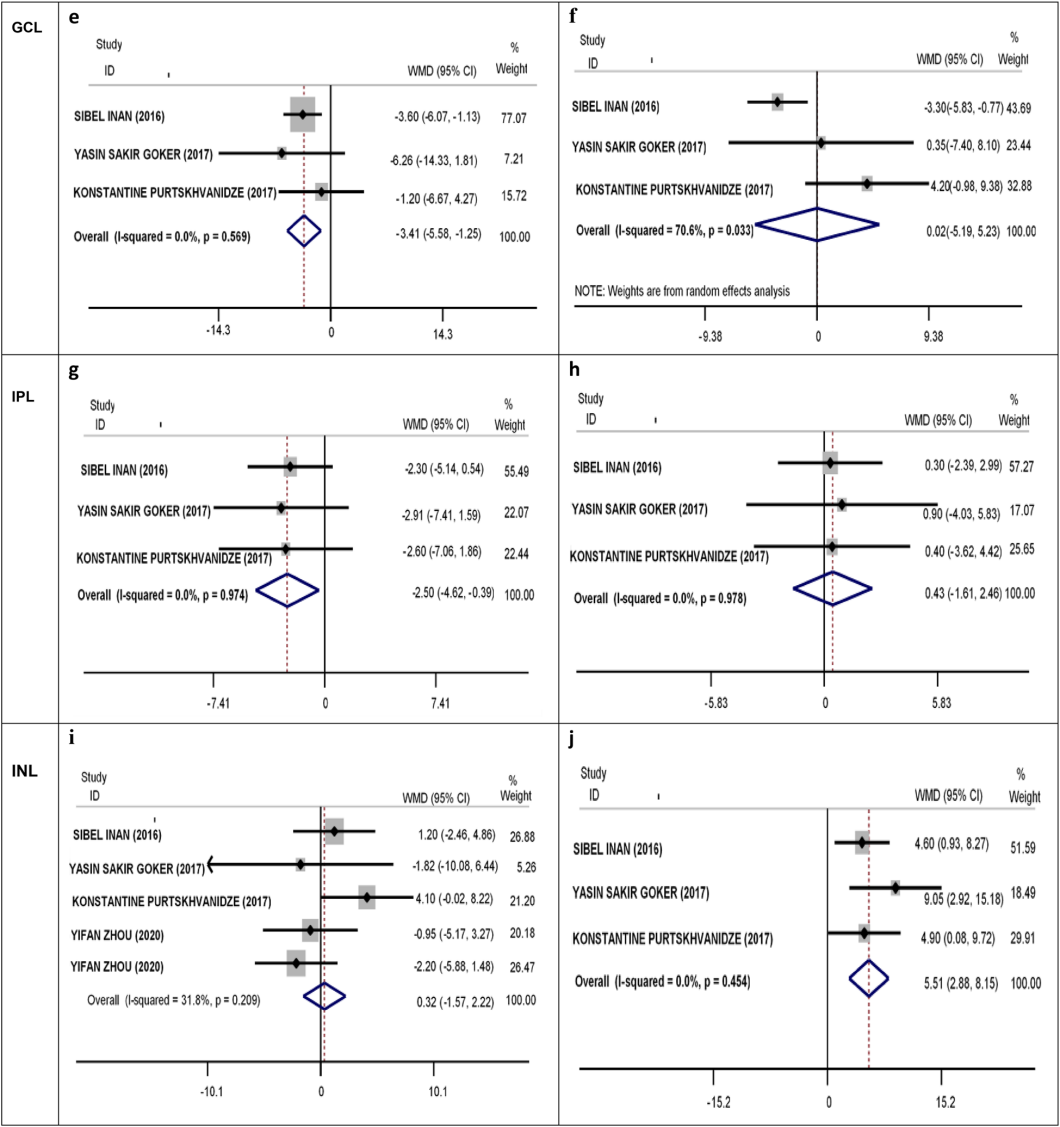

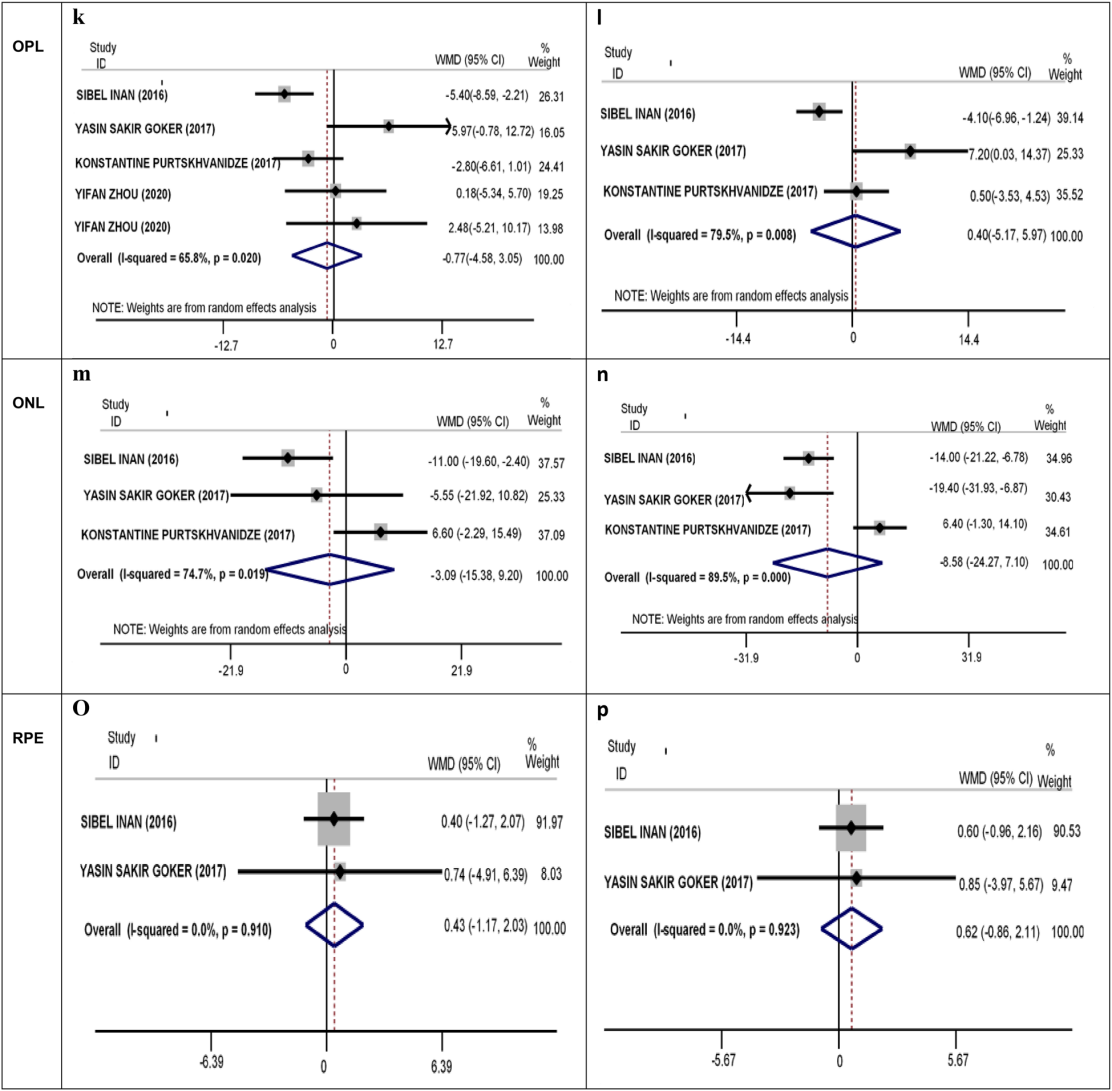
Effect of SO (silicone oil) tamponade on retinal layers in patients with rhegmatogenous retinal detachment (RRD). *SO* silicone oil, *RRD* rhegmatogenous retinal detachment, *CMT* central macular thickness, *NFL* nerve fiber layer, *GCL* ganglion cell layer, *IPL* inner plexiform layer, *INL* inner plexiform layer, *OPL* outer plexiform layer, *ONL* outer nuclear layer, *RPE* retinal pigment epithelium

We examined the effect of SO tamponade on CMT in several sections:

#### Comparison of CMT during SO tamponade application and after SO removal

In this comparison, due to the small number of articles and incomplete information, no meta-analysis was conducted, and the data were only qualitatively examined. This section includes three articles. The results of a study by Hostovsky [24] showed that during SO tamponade application, the CMT was significantly lower, with a thickness recovery after SO removal (from 248 ± 48.88 to 277 ± 46.46 μ; P = 0.001). There was no significant difference in the CMT between the eye with SO tamponade and the fellow eye in the last follow-up after the SO removal (278 ± 49.40 and 264 ± 34.92 μ, respectively; P = 0.265).

Another study by Rabina [27] showed that the mean CMT in the operated eye increased from 249 ± 50 µ during SO tamponade application to 279 ± 48 µ after SO removal (P = 0.001). Also, the CMT was 281 ± 21 µ in the fellow healthy eye. The results showed the apparent thinning of retinal thickness, mainly in the inner layers, during SO tamponade application, which resolved after SO removal. However, a study by Kheir WJ [29] reported that the mean CMT decreased from a baseline of 255.7 µ to 247.5 µ during SO tamponade application, which was followed by an increase to 262.1 µ after SO removal; however, these changes were not statistically significant (P = 0.44). Besides, quantitative thinning of only the inner retinal layers was observed. Based on these three studies, it seems that SO tamponade causes a transient decrease in the CMT, mainly in the inner layers. After SO removal, the CMT resembled that of the fellow eye.

#### Comparison of CMT after SO removal with CMT after gas tamponade (Fig. 2a)

Four studies with five effect sizes [14, 15, 17, 30] examined the effect of SO tamponade on the CMT after SO removal compared to gas tamponade in patients with RRD. The SO tamponade could significantly reduce the CMT (WMD = − 14.91; 95% CI: − 22.23, − 7.60; P < 0.001, I^2^ = 71%) compared to gas tamponade in patients with RRD, with substantial heterogeneity between studies (Fig. 2a). The meta-regression analysis showed that the outcomes were not dependent on age (P = 0.632) or the time spent after SO removal (P = 0.328). However, they were dependent on the duration of SO tamponade application (P = 0.026). Although Egger’s (P = 0.094) and Begg’s (P = 0.221) regression tests did not show significant publication bias, substantial asymmetry was seen in the funnel plot. Accordingly, we performed a trim-and-fill analysis and found that the CMT reduced significantly in the SO tamponade group compared to the gas tamponade group (WMD = − 14.91; 95% CI: − 22.23, − 7.60; P < 0.001, I^2^ = 71%). The sensitivity analysis showed that exclusion of a study did not affect the results obtained.

Another study by Christensen UC, comparing the SO tamponade group with the gas tamponade group, was not included in our meta-analysis for two reasons. First, in this study, only the inner retinal layers were examined. Second, the unit of measurement was reported in pixels, without mentioning the standard deviation. In this study, thinning of the inner retinal layers was observed in the SO-operated eyes (5148 pixels) compared to gas-operated eyes (6897 pixels) (P = 0.002) [8].

#### Comparison of CMT after SO removal with the fellow healthy eye (Fig. 2b)

Six studies [14, 24, 25, 27, 30, 31] examined the effect of SO tamponade on CMT in the eyes of patients with RRD compared to the fellow healthy eyes. No significant change was found in the eyes with SO tamponade (after SO removal) as compared to the fellow healthy eyes regarding CMT (WMD = − 3.52; 95% CI: − 17.63, 10.59; I^2^ = 68.6%), with substantial heterogeneity between studies (Fig. 2b). The meta-regression analysis showed that the results were not dependent on age (P = 0.214), the time spent after SO removal (P = 0.346), or duration of SO tamponade application (P = 0.992). Although Egger’s (P = 0.338) and Begg’s (P = 0.060) regression tests did not show significant publication bias, substantial asymmetry was observed in the funnel plot. The adjusted value, based on the trim-and-fill method, showed no significant change in the eyes with SO tamponade (after SO removal) in comparison with the fellow healthy eyes (WMD = − 7.83; 95% CI = − 22.15, 6.48; P = 0.284). The sensitivity analysis showed that exclusion of a study did not affect the results obtained.

#### Comparison of the inner retinal layer thickness between eyes with SO tamponade and the healthy fellow eyes

This section includes three studies, with two studies only examining the inner retinal layer thickness and one study examining CMT, which was not included in our meta-analysis due to heterogeneity. In the first study by Tode [28], significant thinning of foveal and parafoveal nerve fibers, ganglion cells, and inner plexiform layer (IPL) was observed in the affected eyes (mean: 58.3 ± 13 μm) as compared to the healthy fellow eyes (mean: 84.5 ± 12.3 μm) (P < 0.01) [28]. Moreover, in a study by Caramoy, the inner retinal layers became thinner after the use of SO-based endotamponade (retinal layer volume: 1.127 ± 0.160 mm^3^) compared to the fellow healthy eye (1.363 ± 0.150 mm^3^) (P = 0.012) [34]. Besides, the results of these two studies showed a decrease in the inner retinal layers in eyes with SO tamponade as compared to the fellow healthy eyes. Moreover, a study by Roohipoor et al. showed that CMT was significantly reduced after SO tamponade removal compared to the fellow healthy eye (P = 0.002) [26].

### Effect of SO tamponade on retinal layer segmentation

In this section, the effect of SO tamponade on different layers of the retina was compared after SO removal with gas tamponade and the fellow healthy eye.

#### Nerve fiber layer (NFL) (Fig. 2c, d)

Four studies with five effect sizes [14, 17, 30, 31] examined the effect of SO tamponade on NFL in patients with RRD after SO removal compared to gas tamponade. No significant change was found in NFL between SO tamponade (after SO removal) and gas tamponade in patients with RRD (WMD = − 0.92; 95% CI: − 2.11, 0.27; I^2^ = 12.6%), and there was no heterogeneity between studies (Fig. 2c). Although Egger’s (P = 0.068) and Begg’s (P = 0.066) regression tests did not show significant publication bias, substantial asymmetry was observed in the funnel plot. The adjusted value, based on the trim-and-fill method, showed no significant change in the effect of SO tamponade on NFL in patients with RRD after SO removal compared to gas tamponade (SMD = − 0.220; 95% CI = − 0.625, 0.185; P = 0.287). The results of Egger’s (P = 0.068) and Begg’s (P = 0.066) regression tests revealed no evidence of publication bias. The sensitivity analysis showed that exclusion of a study did not affect the results obtained.

Three studies [14, 30, 31] examined the effect of SO tamponade on NFL in patients with RRD compared to the fellow healthy eye. The SO tamponade could significantly increase NFL in patients with RRD compared to the fellow healthy eye (WMD = 1.86; 95% CI: 0.65, 3.06; I^2^ = 0.0%); there was no heterogeneity between studies (Fig. 2d). The results of Egger’s (P = 0.560) and Begg’s (P = 1.000) regression tests revealed no evidence of publication bias. The sensitivity analysis showed that exclusion of a study did not affect the results obtained.

#### Ganglion cell layer (GCL) (Fig. 2e, f)

Three studies [12, 27, 28] examined the effect of SO tamponade on GCL in comparison with gas tamponade and the fellow healthy eyes. The SO tamponade could significantly reduce GCL in patients with RRD compared to gas tamponade (WMD = − 3.41, 95% CI: − 5.58, − 1.25; I^2^ = 0.0%); there was no heterogeneity between studies (Fig. 2e). Also, no significant change was found in the operated eyes after SO removal (WMD = 0.02, 95% CI: − 5.19, 5.23; I^2^ = 70.6%) compared to the fellow healthy eyes; there was moderate heterogeneity between studies (Fig. 2f). The results of Egger’s and Begg’s regression tests revealed no evidence of publication bias in comparison with gas tamponade (Egger’s test P = 0.589 and Begg’s test P = 1.000) and the fellow healthy eyes (Egger’s test P = 0.296 and Begg’s test P = 0.629). The sensitivity analysis showed that exclusion of a study did not affect the results obtained.

#### Inner plexiform layer (IPL) (Fig. 2g, h)

Three studies [12, 27, 28] examined the effect of SO tamponade on IPL in comparison with gas tamponades and the fellow healthy eyes. The SO tamponade could significantly reduce the IPL in patients with RRD as compared to gas tamponade (WMD = − 2.50, 95% CI: − 4.62, − 0.39; I^2^ = 0.0%) (Fig. 2g). No significant change was found between the eyes with SO tamponade after SO removal (WMD = − 0.43; 95% CI: − 1.61, 2.46; I^2^ = 0.0%) and the fellow healthy eyes (Fig. 2h); there was no heterogeneity between studies. The results of Egger’s and Begg’s regression tests revealed no evidence of publication bias in comparison with gas tamponade (Egger’s test P = 0.803 and Begg’s test P = 1.000) and the fellow healthy eyes (Egger’s test P = 0.603 and Begg’s test P = 0.296). The sensitivity analysis showed that exclusion of a study did not affect the results obtained.

#### Inner nuclear layer (INL)

Four studies with an effect size of five [14, 17, 30, 31] examined the effect of SO tamponade on NFL in patients with RRD after SO removal compared to gas tamponades. No significant change was found in INL between the eyes with SO tamponade (after SO removal) and gas tamponade in patients with RRD (WMD = 0.32; 95% CI: − 1.57, 2.22; I^2^ = 31.8.0%); there was no heterogeneity between studies (Fig. 2i). The results of Egger’s test (P = 0.279) and Begg’s test (P = 0.221) revealed no evidence of publication bias.

Three studies [12, 28, 29] examined the effect of SO tamponade on INL in patients with RRD compared to the fellow healthy eyes. The SO tamponade could significantly increase the INL in patients with RRD compared to the fellow healthy eyes (WMD = 5.51; 95% CI: 2.88, 8.15; I^2^ = 0.0%); there was no heterogeneity between studies (Fig. 2j). The results of Egger’s (P = 0.629) and Begg’s (P = 1.000) regression tests revealed no evidence of publication bias. The sensitivity analysis showed that exclusion of a study did not affect the results obtained.

#### Outer plexiform layer (OPL) (Fig. 2I)

Four studies with an effect size of five [12, 15, 28, 29], as well as three studies [12, 28, 29], examined the OPL thickness changes after SO removal in comparison with gas tamponade and the fellow healthy eyes, respectively. No significant change was found in OPL after SO removal compared to gas tamponade (WMD = − 0.77; 95% CI: − 4.58, 3.05; I^2^ = 65.8%), with substantial heterogeneity between studies (Fig. 2k), or with the fellow healthy eyes (WMD = 0.40; 95% CI: − 5.17, 5.97; I^2^ = 79.5%), with substantial heterogeneity between studies (Fig. 2l). The results of Egger’s and Begg’s regression tests revealed no evidence of publication bias in comparison with gas tamponade (Egger’s test P = 0.224 and Begg’s test P = 0.221) and the fellow healthy eyes (Egger’s test P = 0.147 and Begg’s test P = 0.296). The sensitivity analysis showed that exclusion of a study did not affect the results obtained.

#### Outer nuclear layer (ONL) (Fig. 2m, n)

Three studies [12, 28, 29] examined the ONL thickness changes after SO removal in comparison with gas tamponade and the fellow healthy eyes. No significant change was found in the ONL after SO removal, either compared to gas tamponade (WMD = − 3.09; 95% CI: − 15.38, 9.20; I^2^ = 74.7%) (Fig. 2m) or the fellow healthy eyes (WMD = − 8.58; 95% CI: − 24.27, 7.10; I^2^ = 89.5%), with substantial heterogeneity between studies (Fig. 2n). Comparison of SO tamponade with gas tamponade after removal by Egger’s (P = 0.502) and Begg’s (P = 1.000) regression tests revealed no evidence of publication bias. In comparison of SO removal with the fellow healthy eyes, although the results of Egger’s (P = 0.758) and Begg’s (P = 1.000) regression tests did not indicate significant publication bias, substantial asymmetry was seen in the funnel plot. The adjusted value, based on the trim-and-fill method, showed no significant change (SMD = − 0.483; 95% CI = − 1.440, 0.475; P = 0.323). The sensitivity analysis showed that exclusion of a study did not affect the results obtained.

#### Retinal pigment epithelium (RPE) (Fig. 2o)

Two studies [14, 31] examined the RPE thickness changes after SO removal compared to gas tamponade and the fellow healthy eye. No significant change was found in the RPE after SO removal, either compared to gas tamponade (WMD = 0.43; 95% CI: − 1.17, 2.03; I^2^ = 0.0%) (Fig. 2o) or the fellow healthy eyes (WMD = 0.62; 95% CI: − 0.86, 2.11; I^2^ = 0.0%); there was no heterogeneity between studies (Fig. 2p). Publication bias was not reviewed due to the small number of studies.

### Effect of SO tamponade on SFCT (Fig. 3a–c)

**Fig. 3:**
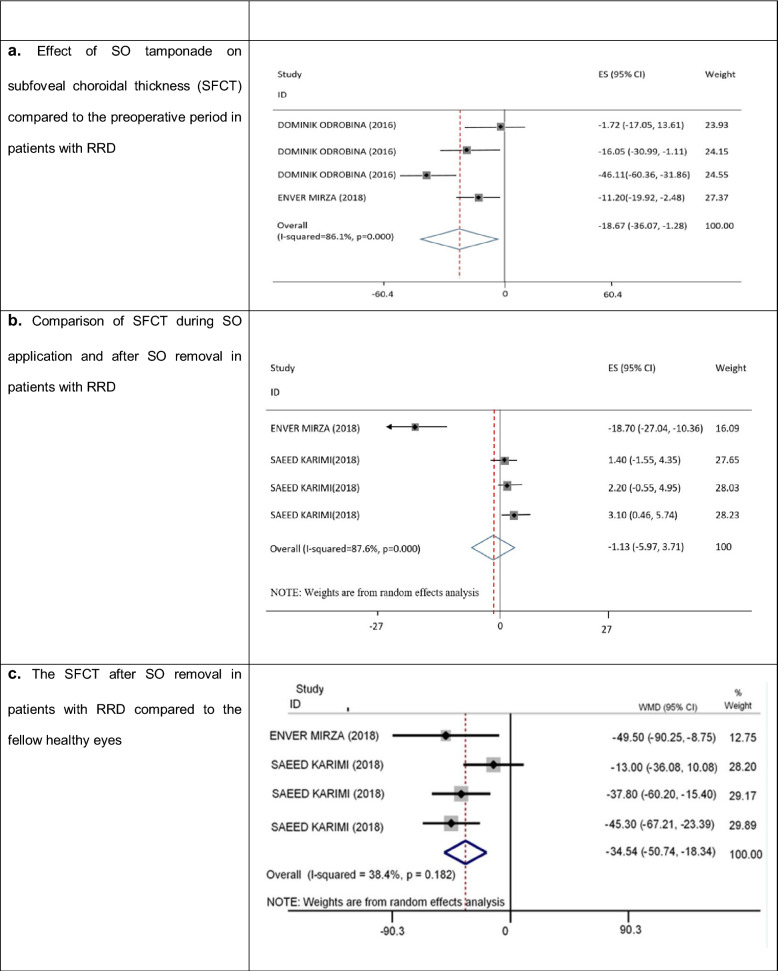
Effect of SO tamponade on SFCT in patients with RRD. *SO* silicone oil, *RRD* rhegmatogenous retinal detachment, *SFCT* subfoveal choroidal thickness

There were three studies regarding the effect of SO tamponade on SFCT, which are described in the following three sections.

#### Comparison of SFCT before and during SO tamponade application (Fig. 3a)

Two studies with an effect size of four [32, 33] examined the effect of SO tamponade on SFCT and compared the results before and during SO application. Compared to the preoperative period, the SO tamponade could significantly reduce SFCT in patients with RRD (WMD = − 18.67; 95% CI: − 30.07, − 1.28; I^2^ = 80.1%), with substantial heterogeneity between studies (Fig. 3a). The meta-regression analysis showed that the results were not dependent on age (P = 0.743) or duration of SO tamponade application (P = 0.730). Although Egger’s (P = 0.711) and Begg’s (P = 0.734) regression tests did not show significant publication bias, substantial asymmetry was observed in the funnel plot. Accordingly, we used the trim-and-fill analysis method and found that the SFCT reduced significantly in the eyes with SO tamponade compared to the preoperative period (WMD = − 24.077; 95% CI = − 41.360, − 6.795; P = 0.006). The sensitivity analysis showed that exclusion of a study did not affect the results obtained.

#### Comparison of SFCT during SO tamponade application and after SO removal (Fig. 3b)

Two studies with an effect size of four [16, 32] examined and compared the SFCT changes during and after SO application. No significant change was found in the SFCT after SO removal, compared to the time of SO tamponade application (WMD = − 1.13, 95% CI: − 5.97, 3.71; I^2^ = 87.6%); there was substantial heterogeneity between studies (Fig. 3b). Our meta-regression analysis showed that the results were not dependent on age (P = 0.039) or duration of SO tamponade application (P = 0.662). The results of Egger’s regression test (P = 0.002) and the funnel plot revealed evidence of publication bias, but the adjusted value, based on the trim-and-fill method, showed no significant change after SO removal compared to the time when SO was removed (WMD = − 3.711; 95% CI = − 10.045, 2.622; P = 0.251). The sensitivity analysis showed that exclusion of a study did not affect the results obtained.

#### Comparison of SFCT after SO tamponade removal with the fellow healthy eyes (Fig. 3c)

Two studies with an effect size of four [32, 33] examined the effect of SO tamponade on the SFCT after SO tamponade removal and compared it with the fellow healthy eyes. After SO removal, the SFCT significantly reduced in patients with RRD compared to the fellow healthy eyes (WMD = − 34.54; 95% CI: − 50.74, − 18.34; I^2^ = 38.4%); there was no heterogeneity between studies (Fig. 3c). The meta-regression analysis showed that the results were not dependent on age (P = 0.855) or duration of SO tamponade application (P = 0.716). Although the results of Egger’s (P = 0.694) and Begg’s (P = 0.308) regression tests did not show significant publication bias, substantial asymmetry was observed in the funnel plot. The adjusted value, based on the trim-and-fill method, showed that the SFCT reduced significantly after SO removal compared to the fellow healthy eyes (VMD = − 34.54; 95% CI: − 50.74, − 18.34; P < 0.001). The sensitivity analysis showed that exclusion of a study did not affect the results obtained.

## Discussion

For the first time, the results of this systematic review and meta-analysis revealed that in patients with RRD, the SO tamponade reduced CMT, and after SO removal, the retinal layer thickness almost returned to its range before surgery. Also, the SO tamponade in these patients reduced SFCT, which remained reduced following the SO removal. Overall, the SO tamponade seems to reduce CMT. In this regard, studies by Hostovsky (P = 0.001) and Rabina (P = 0.001) showed that the SO tamponade caused a significant reduction in CMT, which returned to the normal range after SO removal [24, 27]. Another study by Kheir WJ, (P = 0.44) showed that CMT decreased when the SO tamponade was applied, but increased after SO removal; however, these changes were not significant [29]. Moreover, in two studies by Tode et al. and Caramoy et al., the thickness of the inner retinal layers significantly reduced in the presence of SO tamponade as compared to the fellow healthy eyes [28, 34].

Previous studies have revealed that CMT decreases significantly in the presence of SO tamponade, especially in the inner retinal layers. The causes of retinal thinning associated with the use of SO tamponade have not been fully elucidated. However, several factors may be involved in this phenomenon. One of these factors is the mechanical stress of SO tamponade on the retinal layers in the macula [35]. Among other mechanisms, the subretinal migration of SO may induce inflammation, resulting in the thinning of the retina [36, 37]. Also, emulsified SO may induce an internal limiting membrane defect and be toxic to the retina, causing retinal thinning when entering the intraretinal space [38]. Another explanation for the thinning of the retina in the presence of SO tamponade is that the hydrophobic SO tamponade replaces the natural environment of the hydrophilic vitreous cavity. This waterproofing effect causes retinal dehydration and reduces the thickness of retinal layers, especially the inner layers [27]. Besides, the indirect mechanisms include changes in the concentration of potassium because of failure in potassium siphoning and changes in cytokine levels, which may induce apoptosis and result in the thinning of retinal layers [5].

It seems that CMT is not significantly different between the operated eye and the fellow healthy eye after SO removal. In studies by Rabina et al. [27] and Purtskhvanidze et al. [30, 38], CMT was not significantly different from the fellow healthy eye after SO removal, while in a study by Takker et al. [25], CMT was significantly reduced compared to the fellow healthy eye after SO removal. Our meta-analysis showed that CMT was not significantly different from the fellow healthy eye after SO removal. In other words, the SO tamponade causes a transient decrease in CMT, and after SO removal, CMT is almost similar to that of the fellow healthy eye. Based on this comparison, age, duration of SO application, and time spent after SO removal had no effects on the outcomes [14, 24, 25, 27, 30, 31].

In our meta-analysis, CMT was compared between patients after SO removal and patients with gas tamponade. The results showed that CMT significantly reduced after SO removal compared to gas tamponade. This reduction in CMT was not related to age or the time spent after SO removal, but was related to the duration of SO application; in other words, the longer the SO tamponade was applied, the more CMT was reduced [14, 15, 17, 30]. The decrease in CMT after SO removal, compared to gas tamponade, can be explained by the fact that in some studies on RRD patients with gas tamponade, a long-term increase in NFL, GCL, and IPL was reported compared to the fellow healthy eyes. It was assumed that the retinal nerve fiber layer (RNFL) edema may be a result of surgery itself or may be related to the relative retinal ischemia associated with RRD [22, 35]. On the other hand, in the SO group, these layers showed relative atrophy in the presence of SO tamponade.

It seems that SO induces different effects on different layers of the retina. Inan et al. reported thinning of GCL, OPL, and ONL in the SO group compared to the group with gas tamponade [31]. Moreover, Goker et al. reported that in the gas tamponade group, only INL was significantly increased. In the SO tamponade group, the INL and OPL thicknesses significantly increased, and the ONL thickness significantly decreased compared to the fellow healthy eyes [14]. Purtskhvanidze et al. showed that GCL and IPL were significantly thinner in the SO group compared to the gas tamponade group [30]. Moreover, Lee SH et al. reported that in the SO group, there was a significant decrease in the thickness of all retinal layers, except for the photoreceptor layer after primary RD surgery [15].

In the study of changes in the thickness of different retinal layers after SO removal in comparison with the fellow healthy eye and gas tamponade, the results of our meta-analysis revealed that the thickness of NFL and INL increased significantly in patients after SO removal compared to the fellow healthy eyes, but no significant changes were observed in the GCL, IPL, ONL, OPL, or RPE. On the other hand, in patients with RRD after SO removal, the thickness of GCL and IPL significantly reduced compared to patients for whom gas tamponade was used, while the thickness of NFL, INL, OPL, ONL and RPE did not change significantly.

The increase in NFL thickness after SO removal can be related to surgical NFL edema that may persist for several months after vitrectomy [31]. The increase in INL thickness can be justified by the fact that Müller cells are the radial glial cells of the retina, and their nuclei are located in the INL. The Müller cells play an important role in structural restoration after retinal reattachment; therefore, increasing their activity can increase the thickness of INL [14]. Several studies have reported the degeneration of ganglion cells in the detached retina [39]. In this regard, a rabbit model of RD showed the progressive loss of ganglion cell axons in the detached retina [40]; this may explain the thinning of GCL observed in our meta-analysis.

In the present study, the effect of SO tamponade on SFCT was investigated in three sections. The SFCT showed a significant decrease in the presence of SO tamponade compared to the period before SO injection. However, comparison of SFCT during and after SO tamponade application did not show a significant change. It was also found that SFCT significantly reduced after SO removal compared to the fellow healthy eyes [16, 32, 33]. These findings suggest that in the presence of SO tamponade, SFCT significantly decreases, which persists after SO removal.

To the best of our knowledge, this is the first meta-analysis that review the effect of SO tamponade on the central macular thickness (CMT), different retinal layers, and choroidal thickness.

This study had several limitations. First, only a limited number of articles, investigating the effect of SO tamponade on the retinal layers and choroidal thickness, were included in our meta-analysis. Second, in some of the studies included in this systematic review, only the thickness of the inner retinal layers was reported, and CMT was not mentioned. Also, in some studies examining the retinal layers separately, the sum of two adjacent layers was reported, which in some cases, made it impossible to compare the layers. On the other hand, the small sample size can be an important factor, as it cannot provide us with accurate information. On the other hand, our meta-analysis had its strengths, as it is the first study summarizing the effect of SO tamponade on the retinal layers and choroidal thickness.

## Conclusion

Based on the results, the SO tamponade causes a temporary decrease in CMT, mainly in the inner retinal layers. It also causes a significant reduction in SFCT; however, the mechanisms of these effects on the retina are unclear. It is recommended to conduct further research on more patients with a long-term follow-up.

## Data Availability

Not applicable.
